# Cost-effectiveness analysis of intensity modulated radiation therapy versus robot-assisted radical prostatectomy for patients with low-risk prostate cancer in Japan

**DOI:** 10.1093/jrr/rrag008

**Published:** 2026-03-06

**Authors:** Ataru Igarashi, Keiichi Jingu, Kaoru Ito, Ritsuko Koba, David W Lee

**Affiliations:** Department of Health Economics and Outcomes Research, Graduate School of Pharmaceutical Sciences, The University of Tokyo, 7-3-1 Hongo, Bunkyo-ku, Tokyo, 113-8654, Japan; Department of Health Data Science, Yokohama City University School of Medicine, 3-9-9 Fukuura Kanazawa-ku, Yokohama, Kanagawa 236-0004, Japan; Department of Radiation Oncology, Tohoku University Graduate School of Medicine, 1-1 Seiryo-machi, Aoba-ku, Sendai, Miyagi 980-8574, Japan; Renagence LLC., 2-19-44 Takamatsu, Morioka, Iwate, 020-0114, Japan; Varian Medical Systems, K.K., 1-11-1 Ohsaki, Shinagawa-ku, Tokyo, 141-0032, Japan; Varian Medical Systems, Inc., 801 Pennsylvania Ave, NW Suite 520, Washington, DC, 20004, USA

**Keywords:** prostate cancer, cost-effectiveness analysis, hypofractionated imrt, robot-assisted radical prostatectomy, Japanese healthcare system

## Abstract

This study evaluated the cost-effectiveness of hypofractionated intensity-modulated radiation therapy delivered in 20 fractions (IMRT-20) compared with robot-assisted radical prostatectomy (RARP) for patients with localized low-risk prostate cancer in Japan. A state-transition Markov model was developed from the Japanese healthcare system, using Japanese-specific cost data. Clinical probabilities, adverse event rates and health utility values were primarily derived from international sources, including the ProtecT trial, with extensive sensitivity and scenario analyses to address parameter uncertainty. The base-case analysis compared IMRT-20 and RARP. Scenario analyses included conventional fractionated IMRT (IMRT-38) versus RARP, as well as IMRT-20 versus RARP excluding the utility decrement associated with sexual dysfunction to explore preference-sensitive outcomes. Model outputs included quality-adjusted life-years (QALYs), incremental cost-effectiveness ratios (ICERs) and incremental net monetary benefit (INMB). In the base-case analysis, IMRT-20 yielded a modest QALY gain (0.1164) at slightly higher costs compared with RARP, resulting in an ICER of JPY 143 685 per QALY, well below the Japanese willingness-to-pay threshold of JPY 5 000 000 per QALY. Probabilistic sensitivity analysis showed that IMRT-20 was cost effective in 78.1% of simulations. IMRT-38 was less cost effective because of longer treatment duration and higher resource utilization (ICER JPY 3 317 380 per QALY), although it remained below the threshold. When the disutility of sexual dysfunction was excluded, IMRT-20 was dominated by RARP; however, INMB analysis indicated that IMRT-20 became economically favorable when the disutility exceeded 20%. Overall, IMRT-20 represents a clinically and economically efficient definitive treatment strategy for localized low-risk prostate cancer in Japan, supporting value-based cancer care.

## INTRODUCTION

Prostate cancer is one of the most common malignancies affecting men worldwide, and its incidence continues to increase in many high-income countries. The recent Lancet Commission reported an expected surge in global prostate cancer cases [1]. The Cancer Incidence in Five Continents project also documented this upward trend across developed nations, including Japan [2]. In 2020, ~87 700 new cases of prostate cancer were diagnosed in Japan [3], underscoring the growing burden of this disease in an aging population. The increasing incidence is largely attributed to population aging and the widespread use of prostate-specific antigen (PSA) testing. Many of these newly detected cases involve localized prostate cancer and are classified as low risk, creating the need for careful treatment selection that balances oncological control, quality of life (QOL) and healthcare resource utilization.

Current Japanese clinical practice guidelines recommend several management options for localized prostate cancer, including active surveillance (AS), radical prostatectomy and external beam radiation therapy (EBRT) [4]. Intensity-modulated radiation therapy (IMRT) has become the standard radiation modality because of its superior dose conformality and sparing of adjacent organs, particularly the rectum and bladder [5]. These features translate into favorable long-term functional outcomes, especially for urinary and sexual preservation [6]. In recent years, moderately hypofractionated IMRT regimens have been increasingly adopted in Japan, as they reduce treatment duration while maintaining oncological efficacy. Robust international evidence supporting this approach has been provided by randomized trials of moderate hypofractionation, most notably the CHHiP and PROFIT (Catton CN 2017) trials, which demonstrated the non-inferiority of 20-fraction schedules compared with conventional fractionation [7, 8].

From the surgical standpoint, robot-assisted radical prostatectomy (RARP) has rapidly replaced open prostatectomy in Japan since its inclusion in the national insurance scheme in 2012. RARP is associated with reduced blood loss, shorter hospitalization and faster recovery of urinary continence compared with conventional open approaches [9, 10]. Consequently, RARP has become the dominant surgical technique in many institutions. Nevertheless, both RARP and IMRT are resource-intensive interventions with high upfront costs, which has important implications for Japan’s universal health insurance system. With the rising prevalence of prostate cancer, the economic sustainability of these advanced treatments is a critical concern.

Health economic evaluations, particularly cost-utility analyses, are being increasingly used to inform reimbursement and policy decisions in oncology [11]. Several cost-effectiveness studies have been conducted in Western countries, including analyses based on the ProtecT trial, which compared surgery, radiotherapy, and active monitoring in localized prostate cancer [12, 13]. These studies suggested that radiotherapy offers favorable cost effectiveness under certain assumptions. However, extrapolating these findings to Japan is problematic because of differences in clinical practice patterns, healthcare delivery, reimbursement structures and cultural perceptions of QOL. For example, Japanese patients have been reported to express less distress regarding sexual dysfunction compared with Western populations, which may influence utility assessments and treatment preferences [6].

To address this evidence gap, we conducted a model-based cost-effectiveness analysis comparing IMRT with RARP in patients with localized low-risk prostate cancer in Japan. By incorporating Japanese-specific cost inputs and considering cultural aspects of QOL valuation, this study aimed to generate contextually relevant evidence to support clinical decision-making and health policy development.

## MATERIALS AND METHODS

### Study design and perspective

We conducted a cost-utility analysis comparing hypofractionated IMRT with RARP in patients with localized low-risk prostate cancer. The analysis was performed from the perspective of the Japanese healthcare system, considering only direct medical costs covered by the National Health Insurance program. Both costs and outcomes were discounted at an annual rate of 2% in line with national guidelines for economic evaluation of healthcare technologies [11].

### Model structure

A state-transition Markov model was developed using TreeAge Pro 2021 (TreeAge Software, Inc., Williamstown, MA, USA) to simulate disease progression, treatment outcomes and costs over a 20-year horizon. The model followed a hypothetical cohort of 65-year-old men with D’Amico low-risk prostate cancer. Patients transitioned between five mutually exclusive health states:

- Initial treatment, representing the immediate post-treatment period following IMRT or RARP
- PSA-controlled disease, reflecting maintenance of acceptable PSA levels
- PSA failure (biochemical recurrence), indicating biochemical recurrence
- Uncontrolled disease requiring androgen deprivation therapy (ADT)
- Death (all-cause mortality)

**Fig. 1:**
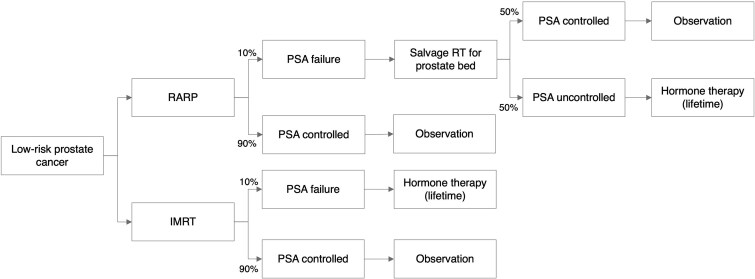
State-transition Markov model simulating post-treatment outcomes of IMRT-20 and RARP in patients with low-risk prostate cancer. Following the initial treatment with either IMRT or RARP, the patients transitioned to either the PSA-controlled state or PSA failure state. In the RARP group, patients with PSA failure received salvage radiation therapy to the prostate bed (IMRT, 66 Gy in 33 fractions), resulting in 50% achieving PSA control and 50% progressing to hormone therapy (leuprorelin plus bicalutamide). By contrast, IMRT-treated patients experiencing PSA failure transitioned directly to hormone therapy without salvage radiation. The model captures the subsequent progression to uncontrolled disease and death. RT, radiation therapy.

At model entry, all patients underwent IMRT or RARP. After initial therapy, 90% were assumed to achieve PSA control, while 10% experienced PSA failure [14, 15]. In the RARP pathway, patients with PSA failure were assumed to receive salvage radiation therapy to the prostate bed (IMRT, 66 Gy in 33 fractions), representing a reference post-recurrence management strategy for non-metastatic biochemical recurrence. Salvage radiotherapy was assumed to restore PSA control in 50% of patients, while the remaining required continuous ADT [16]. This assumption was adopted to reflect guideline-concordant, curative-intent care and to avoid underestimation of downstream treatment costs following surgical management, although not all patients with biochemical recurrence necessarily undergo salvage radiotherapy in real-world practice. By contrast, patients with IMRT failure were assumed to transition directly to ADT without salvage therapy. Monthly cycle lengths were adopted to reflect disease dynamics and treatment sequencing (Fig. 1).

### Treatment strategies

The base-case analysis compared hypofractionated IMRT (60 Gy in 20 fractions; IMRT-20), a regimen widely used in Japan, with RARP. The following two scenario analyses were also conducted:

- Conventional fractionated IMRT (76 Gy in 38 fractions; IMRT-38) versus RARP, to reflect practice variation
- IMRT-20 versus RARP, excluding the utility decrement associated with sexual dysfunction, to explore cultural variation in health state valuation

AS was not included as a comparator. While AS is guideline-recommended for low-risk prostate cancer, the objective of this study was to evaluate cost effectiveness once definitive treatment becomes necessary, as patients who discontinue AS are typically transitioned to surgery or radiotherapy [4, 13].

### Clinical inputs and parameters

Transition probabilities and adverse event (AE) rates were derived from peer-reviewed randomized and observational studies, prioritizing sources reporting long-term QOL outcomes. The PSA control rate was set at 90% for both modalities based on large Japanese and international series [14, 15].

AE rates were primarily derived from the UK-based ProtecT trial, which remains the only randomized study directly comparing surgery and radiotherapy using QOL outcomes prospectively collected for up to 6 years [16]. Although the radiotherapy arm in ProtecT consisted of conventional 3D-conformal EBRT combined with short-term ADT, its use in the present model was justified for several reasons. ADT is known to induce systemic AEs, including sexual dysfunction, which may not be attributable to radiation exposure alone. Therefore, ProtecT-derived toxicity estimates may overestimate early sexual dysfunction associated with contemporary IMRT delivered without ADT. First, compared with the 3D-conformal technique used in ProtecT, modern IMRT achieves superior dose conformality and sparing of organs at risk, while our modeled IMRT cohort did not include ADT. These differences are expected to reduce genitourinary and sexual toxicities, making the ProtecT-based estimates conservative for IMRT.

In contrast, recovery of urinary and sexual function following RARP was modeled based on pooled evidence from contemporary comparative studies, including multi-institutional analyses by Hu *et al.* and Otsuki *et al.* and the meta-analysis by Tang *et al.*, which collectively reported ~65% urinary recovery and 90% sexual recovery within the first postoperative year [9, 10, 17].

Second, this assumption is consistent with external evidence: contemporary Japanese and international IMRT studies have reported favorable urinary and sexual functional outcomes compared with prostatectomy, and recent randomized trials (e.g. CHHiP hypofractionation, NRG-RTOG 0415, PARTIQoL) have demonstrated non-inferior oncologic control with toxicity profiles not worse than historical EBRT [7, 18, 19]. Finally, to address residual uncertainty, all AE parameters informed by ProtecT were varied widely in deterministic and probabilistic sensitivity analyses, ensuring that any potential overestimation of IMRT toxicity was explicitly tested within the model’s uncertainty framework.

All model parameters, including transition probabilities, AE rates and utility decrements, are summarized in Table 1.

**Table 1:** Model input parameters: clinical efficacy, AEs, costs and health utilities for the base-case analysis^a^

Parameter	Base-case value	Source
PSA control ratio		
IMRT	90.0%	Tanaka *et al.* 2018 [14]
RARP	90.0%	Abdollah *et al.* 2016 [15]
Prevalence rate of AEs		
Gastrointestinal symptoms associated with IMRT (3 years)	9.8%	Tanaka *et al.* 2018 [14]
Urinary disorder associated with IMRT (1 year)	3.1%	Lane *et al.* 2022 [16]
Urinary disorder associated with RARP (1 year)	9.8%	Lane *et al.* 2022 [16]
Sexual dysfunction associated with IMRT (1 year)	38.9%	Lane *et al.* 2022 [16]
Sexual dysfunction associated with RARP (1 year)	54.0%	Lane *et al.* 2022 [16]
Cure rate of AEs by RARP		
Urinary disorder (1 year)	64.5%	Tang *et al.* 2017 [17], Hu *et al.* 2014 [9], Otsuki *et al.* 2013 [10]
Sexual dysfunction (1 year)	89.0%	
Medical cost		
IMRT 20 (60 Gy in fractions)	JPY 1 076 180 (USD 7080.1)	Assumptions based on the Medical Fee Schedule in Japan 2024 [20] and the expert’ opinion.
IMRT 38 (76 Gy in fractions)	JPY 1 445 580 (USD 9510.0)	
Surgery cost for RARP	JPY 952 880 (USD 6268.9)	
Hospitalization costs for RARP (estimated LOS:11 days)	JPY 260 880 (USD 1716.3)	
Anesthesia (locoregional anesthesia) for RARP	JPY 100 000 (USD 657.9)	
Salvage IMRT 33 (66 Gy in fractions)	JPY 1 265110 (USD 8323.1)	
Hormonal therapy (annual cost)	JPY 224 690 (USD 1478.2)	
QOL scores (health utilities)		
Base case	0.90	Ramsay *et al.* 2012 [21]
Reduction in QOL due to PSA failure	0.17	Ramsay *et al.* 2012 [21]
Reduction in QOL due to urination disorders	0.17	Lane *et al.* 2022 [16]
Reduction in QOL due to sexual dysfunction	0.11	Lane *et al.* 2022 [16]

^a^USD values were calculated assuming JPY 152 = USD 1. LOS, length of stay.

### Utilities

Effectiveness was measured in quality-adjusted life-years (QALYs). Utility values ranged from 0 (death) to 1 (perfect health). Utility decrements were applied for PSA failure, urinary dysfunction, sexual dysfunction and gastrointestinal toxicity. Utility estimates were primarily drawn from international studies, with supplementation from Japanese data when available [16, 21]. Given the cultural variation in attitudes toward sexual health, we conducted a scenario analysis excluding sexual-dysfunction-related disutility to explore its impact on cost effectiveness.

### Cost estimation

Direct medical costs were estimated using the 2024 Japanese National Fee Schedule, complemented by clinician input to ensure real-world applicability [20]. Components included treatment delivery (planning, simulation and fractions for IMRT; surgery, anesthesia and hospitalization for RARP), management of AEs, salvage therapy and ADT. Routine follow-up and PSA monitoring were assumed to be equivalent across strategies and were therefore excluded. Capital expenditures, such as the acquisition of robotic equipment, were not considered.

All costs were initially calculated in Japanese yen (JPY). For international comparability, costs were converted into US dollars (USD) using the average exchange rate in December 2024 (1 USD = JPY 152).

### Analysis

The total costs and QALYs were accumulated over a 20-year model horizon. The primary outcome was the incremental cost-effectiveness ratio (ICER), calculated as the difference in costs divided by the difference in QALYs between the strategies.


$$ \mathrm{ICER}=\frac{{\mathrm{Cost}}_{\mathrm{IMRT}}-{\mathrm{Cost}}_{\mathrm{RARP}}}{{\mathrm{QALY}}_{\mathrm{IMRT}}-{\mathrm{QALY}}_{\mathrm{RARP}}} $$

An ICER below the cost-effectiveness threshold (i.e. willingness-to-pay threshold, WTP) of 5 000 000 JPY/QALY was considered indicative of cost-effectiveness [11]. Incremental costs and QALYs were estimated as the mean differences between treatment strategies using probabilistic sensitivity analysis (PrSA), thereby capturing the parameter uncertainty across the cohort.

In one-way sensitivity analyses, key parameters (PSA control rates, treatment costs, AE rates and utility values) were varied across plausible ranges or ±25% of base-case values. PrSA was performed with 2 000 000 Monte Carlo iterations. Parameters were assigned beta distributions (probabilities and utilities), gamma distributions (costs) and log-normal distributions (relative risks), based on reported means and standard deviations or confidence intervals [22, 23]. The robustness of the model to cost assumptions in the RARP arm, including downstream treatment costs following biochemical recurrence, was further assessed through these one-way sensitivity analyses.

In scenarios where RARP dominated (less costly and more effective), incremental net monetary benefit (INMB) was calculated using the following formula:

INMB = (Incremental QALYs × Cost-effectiveness threshold) − Incremental Cost.

The INMB is an alternative summary measure that expresses the net economic value of an intervention in monetary terms by directly weighing QALY gains against costs at a given WTP threshold [22, 23]. Unlike the ICER, it remains valid even when one strategy dominates, therefore providing a clinically intuitive measure of whether the additional health benefits of IMRT justify its costs [22, 23]. This method also quantifies the net value of IMRT compared to that of RARP under varying assumptions of disutility from sexual dysfunction (ranging from 0 to 100%), allowing for a culturally sensitive and preference-dependent interpretation of cost-effectiveness.

All base-case and scenario analysis results are presented in Table 2.

**Table 2:** Cost-effectiveness results from base-case and scenario analyses comparing IMRT (20 and 38 fractions) and RARP for low-risk prostate cancer

Interventions	Expected medical costs	Incremental costs	Total expected QALYs	Incremental QALYs	ICER
Base case: IMRT 20 fractions vs. RARP					
IMRT 20 fractions	JPY 1 841 536 (USD 12 115.4)	JPY 16 724 (USD 110.0)	11.5584	0.1164	JPY 143 685 per QALY (USD 945.3 per QALY)
RARP	JPY 1 824 811 (USD 12 005.3)	−	11.4421	−	−
Scenario: IMRT 38 fractions vs. RARP					
IMRT 38 fractions	JPY 2 210 936 (USD 14 545.6)	JPY 386 124 (USD 2540.3)	11.5584	0.1164	JPY 3 317 380 per QALY (USD 21 824.9 per QALY)
RARP	JPY 1 824 811 (USD 12 005.3)	−	11.4421	−	−

## RESULTS

In the base-case analysis, IMRT-20 was not only associated with slightly higher total medical costs but also yielded greater health benefits compared with RARP. The total expected cost was JPY 1 841 536 (USD 12 115.4) for IMRT-20 and JPY 1 824 811 (USD 12 005.3) for RARP, corresponding to an incremental cost of JPY 16 724 (USD 110.0). The expected QALYs were 11.5584 for IMRT-20 and 11.4421 for RARP, resulting in a net gain of 0.1164 QALYs. These differences, though modest in absolute terms, translated into an ICER of JPY 143 685 (USD 945.3) per QALY. This figure is well below the nationally accepted WTP threshold of JPY 5 000 000 per QALY, strongly suggesting that IMRT-20 represents a cost-effective treatment strategy for localized low-risk prostate cancer under the current Japanese healthcare system (Table 2). The results highlight that even a relatively small improvement in long-term quality-adjusted survival can justify a slight increase in upfront treatment cost when evaluated within the framework of value-based cancer care.

**Fig. 2:**
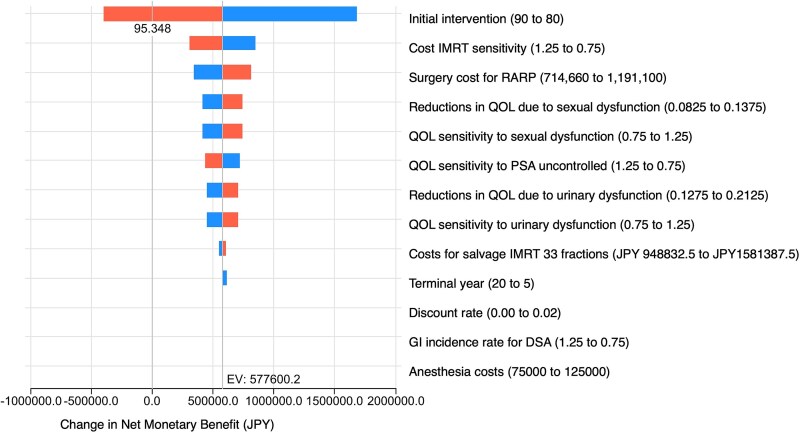
Tornado diagram from one-way sensitivity analysis of the cost-effectiveness model comparing IMRT-20 and RARP. The horizontal bars illustrate the change in the net monetary benefit (NMB, in Japanese yen) when each model parameter is varied between its low and high values while holding all others constant. The vertical dashed line indicates the base-case NMB of JPY 577 600. The parameters were sorted by the magnitude of their impact on the NMB. IMRT cost sensitivity was the most influential factor, followed by surgery cost for RARP and quality-of-life weights related to sexual dysfunction. GI, gastrointestinal symptoms.

The robustness of these findings was confirmed by deterministic sensitivity analyses. As illustrated in the tornado diagram (Fig. 2), the probability of initial PSA control exerted the greatest impact on the INMB of IMRT-20 compared with that of RARP. Reducing the PSA control rate from 90 to 80% generated the largest decrease in economic benefit. The unit cost of IMRT and surgical cost of RARP also substantially influenced the results, reflecting the central role of treatment pricing in shaping cost-effectiveness outcomes. By contrast, changes in utility decrements related to sexual dysfunction, urinary dysfunction and PSA failure exerted only moderate effects on the results. Importantly, across the full range of plausible values tested, INMB remained positive, indicating that IMRT-20 consistently retained economic superiority over RARP. These results underscore that the model’s conclusions are not overly dependent on any single parameter but rather hold under a wide spectrum of clinical and economic assumptions.

**Fig. 3:**
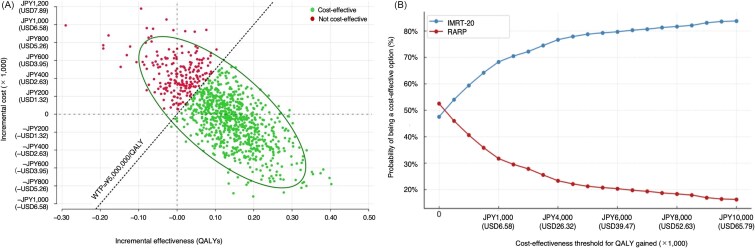
PrSA comparing IMRT-20 and RARP: (A) Incremental cost-effectiveness scatterplot; (B) CEAC. (A) Scatterplot of 2 million Monte Carlo simulations on the cost-effectiveness plane. Each dot represents a single iteration, with green and red indicating cost-effective and not cost-effective outcomes, respectively, based on a WTP of JPY 5 000 000 per QALY (USD 32 900). (B) CEAC showing the probability that each strategy is cost-effective over a range of cost-effectiveness thresholds. IMRT-20 is more likely to be cost effective across commonly accepted WTP values in Japan. US dollar values were calculated assuming JPY 152 = USD 1. CEAC, cost-effectiveness acceptability curve.

The PrSA provided further support for the base-case findings. Based on a large number of (2 000 000) Monte Carlo simulations, the majority of estimates were located in the southeast quadrant of the cost-effectiveness plane, suggesting that IMRT-20 was not only more effective but also frequently less costly than RARP (Fig. 3A). At the commonly accepted WTP threshold of JPY 5 000 000 per QALY, IMRT-20 was found to be cost effective in 78.1% of simulations. This relatively high probability of cost-effectiveness provides confidence that the observed advantage of IMRT-20 is unlikely to be an artifact of model uncertainty. The cost-effectiveness acceptability curve (Fig. 3B) reinforced this interpretation by showing that IMRT-20 maintained a higher likelihood of being the preferred option across a broad range of threshold values, even under more conservative assumptions such as JPY 2 000 000 per QALY.

In the scenario analysis comparing conventional IMRT-38 against RARP, the extended fractionation schedule was found to be less economically favorable. The expected cost of IMRT-38 was JPY 2 210 936 (USD 14 545.6), substantially higher than that of RARP, while the QALY outcome (11.5584) remained identical to that of the 20-fraction regimen. Consequently, the incremental cost reached JPY 386 124 (USD 2 540.3), and the ICER rose to JPY 3 317 380 (USD 21 824.9) per QALY (Table 2). Although this ICER still falls below the Japanese WTP, the larger financial burden associated with IMRT-38 substantially reduces its economic attractiveness. These results emphasize that hypofractionated regimens not only provide equivalent clinical efficacy but also confer a meaningful economic advantage by reducing treatment duration, resource use and patient burden.

**Fig. 4:**
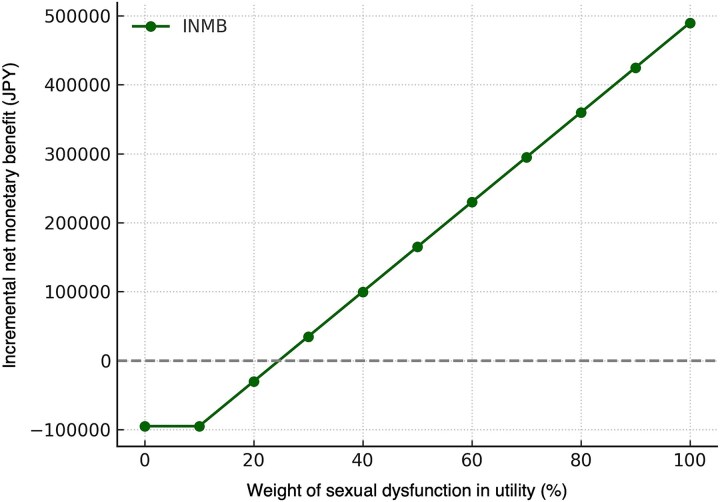
INMB of IMRT-20 compared with RARP across varying utility weights for sexual dysfunction (0–100%). The INMB was plotted against the assigned utility weight for sexual dysfunction, which ranged from 0 to 100%. As the weight assigned to sexual dysfunction increased, the economic value of IMRT improved, with INMB crossing the positive territory at ~20%, indicating that IMRT is the economically preferred strategy beyond this threshold.

A further scenario analysis examined the impact of sexual dysfunction on model outcomes by varying the associated utility decrement from 0 to 100%. When the decrement was assumed to be 0%, IMRT-20 was dominated by RARP, yielding slightly lower QALYs (difference −0.0156) at a higher cost. However, as increasing weight was assigned to the disutility of sexual dysfunction, the relative benefit of IMRT-20 grew proportionally. At 20%, IMRT-20 achieved a modest gain of 0.0104 QALYs, with an ICER of JPY 1 635 553 per QALY, while at 50 and 100%, the QALY gains rose to 0.0494 and 0.1143, respectively, with ICERs decreasing to JPY 148 797 per QALY at full weighting. Because ICER values are undefined in dominance scenarios, INMB was also used as a complementary measure. INMB shifted from −JPY 94 891 at 0% to +JPY 554 491 at 100%, crossing into positive territory at ~20% weighting (Fig. 4, Supplementary Table 1). These findings reveal that the economic value of IMRT-20 depends strongly on how much importance is attached to preserving sexual function. From a clinical perspective, they illustrate how cultural differences in the perception and reporting of sexual health can substantially influence cost-effectiveness conclusions and highlight the necessity of incorporating patient preferences into economic evaluations.

## DISCUSSION

This study evaluated and compared the cost-effectiveness of IMRT-20 and RARP for patients with localized low-risk prostate cancer in Japan. Using a Markov model populated with Japanese-specific cost and utility data, we demonstrated that hypofractionated IMRT delivered in 20 fractions offers a favorable trade-off between clinical benefit and economic value compared with RARP. IMRT-20 achieved a modest QALY gain (0.1164) at only slightly higher costs, resulting in an ICER of JPY 143685 per QALY, which is far below the Japanese WTP of JPY 5 000 000 per QALY. These findings indicate that IMRT-20 is a cost-effective treatment option for localized prostate cancer in the current Japanese healthcare context. The base-case result was robust across one-way sensitivity analyses, in which the probability of initial disease control, treatment costs and utility weights exerted the greatest influence. Moreover, PrSA demonstrated that IMRT-20 was cost effective in 78.1% of simulations, supporting the reliability of our conclusions under real-world uncertainty.

In this study, comparators were limited to IMRT and RARP. AS is widely recognized as the standard management technique for low-risk prostate cancer, and its superior cost-effectiveness is well established [4, 5]. However, in clinical practice, a proportion of patients are unable or unwilling to remain on AS, and definitive treatment with RARP or IMRT is commonly pursued in such cases` [13]. Our analysis was, therefore, designed to provide evidence for decision-making specifically between radical therapies, which remains a relevant scenario in Japan. While future research should incorporate AS into broader models, our current focus provides practical insights for patients and clinicians when definitive treatment is required.

QOL parameters were primarily based on the ProtecT trial [16]. Although it was conducted more than a decade ago and included EBRT + ADT in the radiotherapy arm, ProtecT remains the only randomized controlled trial directly comparing surgery and radiotherapy using long-term prospectively collected patient-reported outcomes [16]. These features make it uniquely valuable for economic evaluations requiring consistent QOL comparisons across modalities. Alternative sources, such as prospective cohort studies of IMRT or registry-based datasets, either lack surgical comparators or provide shorter follow-up, limiting their applicability to our model [6, 24]. Therefore, despite its limitations, the ProtecT trial represents the most robust and methodologically rigorous dataset available for this purpose. Moreover, applying EBRT + ADT toxicities to IMRT without ADT may overestimate early AEs, particularly sexual dysfunction, thereby representing an upper-bound estimate for IMRT-related QOL decrements. Recent prospective studies, including those by Hoffman *et al.* and Namiki *et al.*, suggest that IMRT has limited urinary and sexual toxicity, reinforcing the validity of our approach while underscoring the need for Japan-specific QOL research [6, 24]. Additionally, recent randomized and prospective studies further support the favorable toxicity profile of modern radiotherapy. The NRG RTOG 0415 trial confirmed the non-inferiority of moderate hypofractionation when compared with conventional fractionation in favorable-risk patients, with long-term follow-up [18]. The PARTIQoL trial demonstrated comparable QOL outcomes between proton therapy and IMRT, highlighting the value of contemporary external-beam approaches in preserving patient-reported outcomes [19]. Similarly, Sveistrup *et al.* prospectively assessed QOL before, during and after image-guided IMRT, showing minimal long-term declines in urinary and sexual function [25]. Together, these findings corroborate our assumption that IMRT toxicities can be overestimated when extrapolated from EBRT + ADT cohorts and highlight the need for Japanese-specific longitudinal QOL studies.

Scenario analyses further emphasized the impact of treatment design on economic outcomes. Conventional IMRT, delivered in 38 fractions, was substantially less efficient than IMRT-20, despite equivalent oncological effectiveness, due to higher costs and greater utilization of healthcare resources. These findings are consistent with from randomized trials of moderate hypofractionation, most notably the CHHiP and PROFIT trials, which confirmed the non-inferiority of 20-fraction regimens compared with conventional schedules [7, 8]. In the Japanese context of an aging population and a limited oncology workforce, shorter yet evidence-based regimens may reduce waiting times, improve facility utilization and lessen patient burden, thereby enhancing both system-level efficiency and patient-centered care.

The scenario excluding disutility from sexual dysfunction suggested that RARP was more favorable. This highlights the sensitivity of cost-effectiveness estimates to the valuation of sexual function. Cultural differences are relevant: Japanese men often report less distress from sexual dysfunction than Western populations, and Namiki *et al.* showed that urinary outcomes and oncological control are prioritized over sexual outcomes [6]. Such differences may lead to undervaluation of sexual health in QALY-based models. Through INMB analysis, we demonstrated that even modest weighting of sexual function preservation shifted results in favor of IMRT-20, providing a more nuanced interpretation.

An important consideration in interpreting these results is the exclusion of AS, which is currently recommended as a standard management option for patients with low-risk prostate cancer. In principle, incorporating an AS-first strategy could reduce short-term costs and increase QALYs by delaying treatment-related disutility, and might therefore appear to disadvantage immediate definitive treatment strategies.

However, AS represents a distinct decision-making stage whether to initiate curative treatment at all, whereas the present analysis was intentionally designed to compare definitive treatment strategies once that decision has been made. Importantly, patients transitioning from AS would ultimately receive either IMRT or RARP, such that the relative comparison between definitive modalities remains the primary determinant of incremental cost-effectiveness. Moreover, because AS primarily delays the onset of treatment-related harms, its inclusion may disproportionately benefit surgical strategies characterized by greater upfront invasiveness and early quality-of-life decrements. Consequently, exclusion of AS is more likely to underestimate, rather than overestimate, the relative economic value of non-surgical definitive treatments such as IMRT. While future models explicitly incorporating AS would be valuable, we believe that its exclusion does not materially bias the present findings toward overstating the superiority of IMRT over RARP.

Our findings are consistent with those of previous economic studies conducted in other countries. For example, Sanghera *et al.* reported that radiotherapy was the most cost-effective treatment strategy for localized prostate cancer in the UK [12]. Similarly, Noble *et al.* performed a cost-effectiveness analysis using the ProtecT trial and found that radiotherapy was economically favorable compared with surgery or active monitoring [13]. Other modeling studies, such as those by Carter *et al.* in Australia and Zemplényi *et al.* in Hungary, also concluded that IMRT, particularly in its hypofractionated form, has high economic value [26, 27]. These results highlight the generalizability of our findings while underscoring the importance of incorporating Japanese-specific cost structures and cultural contexts.

Importantly, our scenario analysis revealed that conventional fractionated IMRT (IMRT-38) was less cost effective than IMRT-20, with an ICER of JPY 3 317 380/QALY. Although still below Japan’s accepted threshold, the extended treatment duration and associated costs reduced its economic attractiveness. This aligns with evidence from the CHHiP trial and other studies showing comparable outcomes between ultra-hypofractionated and conventional regimens [7]. Internationally, the PACE-B trial also demonstrated that SBRT achieved comparable acute toxicity profiles to those of conventional IMRT, further supporting the value of shorter-course regimens, such as IMRT-20 [28]. From a health system perspective, shorter regimens alleviate patient burden, reduce resource utilization and improve system efficiency—an important consideration for Japan’s aging society and workforce-constrained oncology services.

In the Japanese clinical setting, other definitive radiotherapy modalities, including low-dose-rate brachytherapy (LDR-BT) and SBRT, are also widely used for patients with low-risk prostate cancer. These modalities have demonstrated excellent long-term oncological control comparable to that of IMRT and surgery and are endorsed in domestic clinical guidelines [4].

From an economic perspective, however, their cost-effectiveness profiles differ and should not be considered uniform. LDR-BT has been associated with favorable toxicity profiles, including lower rates of rectal toxicity and relatively good preservation of sexual function in selected patients. Nevertheless, its higher procedural and hospitalization-related costs in the Japanese reimbursement system may limit its overall economic attractiveness when compared with other definitive treatment strategies. In contrast, SBRT delivers comparable tumor control with substantially shorter treatment courses and lower reimbursement costs. Owing to its reduced resource utilization and treatment duration, SBRT may represent a potentially cost-effective alternative among definitive radiotherapy options in Japan, although robust long-term quality-of-life and cost data remain limited.

The present analysis intentionally focused on IMRT and RARP, which are broadly accessible and commonly compared definitive treatment strategies at the point of treatment decision-making in routine clinical practice. Incorporating LDR-BT and SBRT would require substantial expansion of the model structure to account for distinct treatment pathways, toxicity profiles, institutional availability and reimbursement schemes, potentially increasing structural uncertainty. Accordingly, while LDR-BT and SBRT were not included as comparators in the present model, they represent important candidates, particularly SBRT, for future cost-effectiveness analyses within a unified modeling framework tailored to the Japanese healthcare context.

### Limitations

Our study has some limitations. First, this analysis did not include AS, which is a recommended management option for patients with low-risk prostate cancer. This study was intentionally designed to evaluate the cost-effectiveness of definitive treatment strategies once the decision to initiate curative interventions has been made, rather than to compare initial management strategies that include deferred treatment. AS represents a distinct decision-making stage involving different clinical trajectories, timing of intervention and QOL trade-offs. Incorporating AS into the same model would require additional assumptions regarding disease progression, delayed treatment initiation and heterogeneous downstream treatment pathways, which would substantially increase model uncertainty and obscure the primary comparison between IMRT and RARP. Importantly, the exclusion of AS is more likely to underestimate, rather than overestimate, the relative economic value of non-surgical definitive treatments, because AS prolongs the period without treatment-related disutility, particularly those associated with upfront surgical intervention.

Second, although Japanese-specific cost inputs were used, most clinical parameters and utility values were derived from international sources, notably the UK-based ProtecT trial. Cultural differences in healthcare practices, symptom reporting and patient priorities may limit the direct applicability of these data.

Third, the use of the ProtecT trial outcomes may have overestimated IMRT-related toxicity, particularly early sexual dysfunction, because patients in the radiotherapy arm received EBRT combined with short-term ADT, which is not standard in contemporary Japanese practice [16]. ADT is associated with systemic adverse effects that may not be attributable to radiation exposure alone. Although this mismatch in treatment protocols represents a structural limitation of the model, long-term functional outcome data from the ProtecT trial remain among the most robust sources available for comparative modeling. Importantly, this uncertainty was explicitly addressed through extensive sensitivity and scenario analyses varying the disutility associated with sexual dysfunction across a wide range.

Fourth, the model assumed that all patients with biochemical recurrence after RARP received salvage radiotherapy, which may not fully reflect real-world clinical practice, where some patients are managed with observation or systemic therapy alone depending on disease characteristics and patient preferences. Often involving earlier initiation of long-term ADT, which is associated with ongoing costs and QOL impact. This assumption was intentionally adopted as a reference post-recurrence strategy to represent guideline-concordant, curative-intent management of non-metastatic PSA recurrence and to avoid underestimation of downstream treatment costs following surgical intervention. Importantly, one-way sensitivity analyses demonstrated that variation in RARP-related cost parameters had a relatively limited impact on the net monetary benefit, and the overall cost-effectiveness conclusions remained unchanged, suggesting that potential deviations from this assumption are unlikely to materially affect the study findings.

Fifth, advanced systemic therapies such as novel androgen receptor signaling inhibitors (ARSIs) were not included in the present model. In patients with localized low-risk prostate cancer, progression to castration-resistant prostate cancer is relatively uncommon and typically occurs late in the disease course, beyond the period in which initial local treatment strategies primarily influence costs and quality-adjusted survival. Although patients receiving long-term ADT, particularly those in the IMRT arm, may theoretically incur higher downstream costs if ARSIs are introduced, such costs would apply only to a small subset of patients and are unlikely to materially alter the relative cost-effectiveness comparison between IMRT and RARP, given the modest differences in overall survival observed in the model. Future studies focusing on higher-risk populations or longer lifetime horizons may benefit from explicitly incorporating advanced systemic therapies as more robust Japan-specific data on treatment sequencing and real-world utilization become available.

Finally, several modeling and scope-related limitations should be noted. Opportunity costs associated with extended treatment schedules (e.g. staff time, equipment uses) were not explicitly quantified in the model. In addition, emerging technologies, such as MR-guided RT and proton therapy, were not included but may influence future cost-effectiveness outcomes.

In conclusion, hypofractionated IMRT delivered in 20 fractions represents a clinically effective and economically efficient treatment strategy for localized low-risk prostate cancer in Japan. It demonstrated superior cost-effectiveness when compared with both conventional IMRT and RARP, particularly when resource constraints and patient-centered outcomes are considered. These findings support the integration of IMRT-20 into Japanese clinical guidelines and reimbursement frameworks and highlight the need for Japan-specific prospective QOL studies to refine culturally relevant value assessments.

## Supplementary Material

6_Supplementary_Table_1_rrag008

## PRESENTATION AT A CONFERENCE

Preliminary results of this study were presented at the 2021 Annual Meeting of the Japanese Society for Radiation Oncology (JASTRO 2021). The current analysis includes updated data, expanded model inputs and additional scenario analysis.
