# Correction to: Cost-effectiveness analysis of intensity modulated radiation therapy versus robot-assisted radical prostatectomy for patients with low-risk prostate cancer in Japan

**DOI:** 10.1093/jrr/rrag065

**Published:** 2026-07-16

This is a correction to: Ataru Igarashi, Keiichi Jingu, Kaoru Ito, Ritsuko Koba, David W Lee, Cost-effectiveness analysis of intensity modulated radiation therapy versus robot-assisted radical prostatectomy for patients with low-risk prostate cancer in Japan, *Journal of Radiation Research*, Volume 67, Issue 2, March 2026, Pages 266–275, https://doi.org/10.1093/jrr/rrag008

After publication, the authors identified errors in the description of the radiation modality in two places in the article.

Specifically, under “Model structure” in the Materials and Methods section and in the Figure 1 caption, the modality was incorrectly described as “3D-CRT,” whereas the correct modality is “IMRT.”

This change has been reviewed by the Editor-in-Chief, who agrees with the authors’ assertions that it does not affect the analyses, results, or conclusions of the study.

