# Supplementary material for: Cost-effectiveness analysis of intensity modulated radiation therapy versus robot-assisted radical prostatectomy for patients with low-risk prostate cancer in Japan
Source: J Radiat Res. 2026 Mar 6;67(2):266–75. doi: 10.1093/jrr/rrag008 (PMC13019139; doi:10.1093/jrr/rrag008)
Supplement: 6_Supplementary_Table_1_rrag008 [file 6_supplementary_table_1_rrag008.docx]

Supplementary Table 1. Impact of sexual dysfunction utility weight on ICER and INMB (IMRT vs. RARP)

| Sexual Dysfunction Weight (%) | ICER  (JPY/QALY) | INMB (IMRT vs. RARP) (JPY) |
| --- | --- | --- |
| 0 | Dominated | −94,891 |
| 10 | Dominated | −29,953 |
| 20 | 1,635,553.1 | 34,986 |
| 30 | 727,243.1 | 99,924 |
| 40 | 467,574.2 | 164,862 |
| 50 | 344,549.6 | 229,800 |
| 60 | 272,778.2 | 294,738 |
| 70 | 225,752.8 | 359,676 |
| 80 | 192,557.0 | 424,615 |
| 90 | 167,872.3 | 489,553 |
| 100 | 148,797.3 | 554,491 |

IMRT, intensity-modulated radiation therapy; RARP, robot-assisted radical prostatectomy; ICER, incremental cost-effectiveness ratio; INMB, incremental net monetary benefit; QALY, quality-adjusted life year; JPY, Japanese yen
